# Genetic variants, pathophysiological pathways, and oral anticoagulation in patients with hypertrophic cardiomyopathy and atrial fibrillation

**DOI:** 10.3389/fcvm.2023.1023394

**Published:** 2023-04-17

**Authors:** Shengnan Wang, He Chen, Chunju Liu, Mengxian Wu, Wanlei Sun, Shenjian Liu, Yan Zheng, Wenfeng He

**Affiliations:** ^1^ Department of Medical Genetics, The Second Affiliated Hospital of Nanchang University, Nanchang, China; ^2^ Jiangxi Key Laboratory of Molecular Medicine, The Second Affiliated Hospital of Nanchang University, Nanchang, China; ^3^ Department of Clinical Laboratory, Affiliated Hospital of Jiangxi University of Traditional Chinese Medicine, Nanchang, China; ^4^ Department of Clinical Laboratory, The Second Affiliated Hospital of Nanchang University, Nanchang, China

**Keywords:** atrial fibrillation, hypertrophic cardiomyopathy, genotype, mechanism, anticoagulation

## Abstract

Atrial fibrillation (AF) is commonly prevalent in patients with hypertrophic cardiomyopathy (HCM). However, whether the prevalence and incidence of AF are different between genotype-positive vs. genotype-negative patients with HCM remains controversial. Recent evidence has indicated that AF is often the first presentation of genetic HCM patients in the absence of a cardiomyopathy phenotype, implying the importance of genetic testing in this population with early-onset AF. However, the association of the identified sarcomere gene variants with HCM occurrence in the future remains unclear. How the identification of these cardiomyopathy gene variants should influence the use of anticoagulation therapy for a patient with early-onset AF is still undefined. In this review, we sought to assess the genetic variants, pathophysiological pathways, and oral anticoagulation in patients with HCM and AF.

## Introduction

Atrial fibrillation (AF) is commonly seen in patients with hypertrophic cardiomyopathy (HCM). The estimated prevalence of AF in HCM patients is 22.3%, and the incidence of AF is 2.5 cases per person-years (1). Genes encoding thick-filament (MYH7, MYBPC3; nearly 40%) and thin-filament (TNNT2, TNNI3, TPM1, TNNC1, ACTC1; nearly 5%) proteins are the two most common types associated with HCM development, followed by other sarcomeric genes (e.g., TTN, MYL2, MYL3, OBSCN, TRIM63, JHP2, ACTN2, CRSP3, MYZO2) (2). Recent evidence has suggested that AF is often the first presentation of genetic HCM patients in the absence of a cardiomyopathy phenotype, implying the importance of genetic testing in this population with early-onset AF. However, the association of the identified sarcomere gene variants with HCM occurrence in the future remains unclear. In this review, we aimed to assess the genetic variants, pathophysiological pathways, and oral anticoagulation in patients with HCM and AF.

## Prevalence and incidence of AF in inherited HCM patients

AF is the most common persistent cardiac arrhythmia disease, affecting 1% to 4% of the general population, which leads to an increased risk of heart failure and stroke, further causing substantial morbidity and mortality. More recently, a growing body of evidence has found that some gene variants accounting for inherited cardiomyopathies, such as dilated cardiomyopathy (DCM), HCM, and arrhythmogenic right ventricular cardiomyopathy (ARVC), are also primarily related to substantial AF penetrance (3).

In the study of Butters et al., the incidence of AF in hereditary cardiomyopathy was HCM (31%), left ventricular noncompaction cardiomyopathy (LVNC) (18%), DCM (17%), and ARVC (14%) (3). Bongini et al. analysed the prevalence and clinical correlates of AF in relation to genotype and evaluated 237 patients with HCM followed for 14 ± 10 years. AF occurred in 74 patients with HCM (31%), with no differences among groups (31% in MYBPC3, 37% in MYH7% and 18% in other genotypes) (4). Akhtar et al. recruited 537 TTN gene carriers with truncating variants (TTNtv) in DCM and followed them for a median of 49 (18–105) months. In the final assessment, 31% of the patients had AF (5). Currently, only a few dozen patients with SCN5A-mediated cardiomyopathies have been described. Mutations are associated with DCM, ARVC, and atrial standstill. These cardiomyopathies are usually characterized by a wide range of rhythm disorders. The incidence of AF with SCN5A reported by Zaklyazminskaya was 40%–60% (6). However, there are no data on the incidence of AF in different hereditary cardiomyopathies in the same type of gene mutation. Currently, most data describing the prevalence and impact of AF in patients with cardiomyopathy have focused on HCM, with limited data describing patients with LVNC, DCM or ARVC.

The prevalence of AF in HCM has been found to be not different between genotype-positive vs. genotype-negative patients in previous studies (7–11). Among these studies, Olivotto et al. (7) reported that the prevalence of paroxysmal or chronic AF in HCM patients at baseline was independent of the genetic background. However, when chronic AF was independently analysed, it was more prevalent in genotype-positive patients with HCM than in genotype-negative controls. Moreover, the AF prevalence and baseline left atrial diameter were comparable between thin-filament and thick-filament mutation-associated HCM patients (12). In the study of Bongini et al. (4), the studied HCM population was divided into three genotypes, namely, MYBPC3 (58%), MYH7 (28%), and other sarcomeric genes (14%), suggesting no differences in the prevalence of paroxysmal/persistent AF, paroxysmal evolved to permanent AF, or chronic AF only among the three subgroups.

A meta-analysis of 51 studies with 7,675 HCM individuals found that 18% of HCM patients with MYBPC3 variants, 24% of MYH7, 33% of TNNT2, 30% of TNNI3, and 17% of genotype-negative (no pathogenic variants in sarcomere genes) patients showed supraventricular tachycardia, such as AF. Lee et al. (13) found that 19% of HCM patients with sarcomeric gene variants developed new-onset AF. In the Genotyped HCM Cohort (14), when compared with genotype-negative patients with HCM, sarcomere mutation carriers (pathogenic or likely pathogenic variant [hazard ratio (HR) = 2.41, 95% confidence interval (CI): 1.98–2.94], sarcomere variant of unknown significance at present [HR = 1.90, 95% CI: 1.38–2.64]) had an increased risk of incident AF after controlling for proband status, sex, and race. However, the AF risk was similar regardless of whether the sarcomere variant was pathogenic or of unknown significance at present (14), suggesting that these variants of unknown significance in sarcomere genes could affect the HCM prognosis. In the follow-up, the genetic subtype was seemingly not an independent predictor of new-onset AF (thin vs. thick-filament: 11% vs. 9%, *P* = 0.527 [Coppini et al.] and MYBPC3 vs. MYH7 vs. other genotypes: 31% vs. 37% vs. 18%, *P* = 0.15 [Bongini et al.]) in HCM patients (4, 12). Of note, in the study by Bongini et al. (4), HCM patients with MYH7 variants had a higher risk of AF than other genotypes, although the difference was not significant. Indeed, a subsequent study by Lee et al. (13) further demonstrated that HCM patients with likely pathogenic or pathogenic MYH7 variants had a higher risk of incident AF than other sarcomeric genes (MYBPC3, thin filament genes). In addition, patients with sarcomeric gene (e.g., MYH7, TNNT2) variants in hot spot sites that are more frequently associated with HCM development may have higher AF vulnerability in the future than those with gene variants in non-hot spot sites (13, 15).

## Cardiomyopathy gene variants and early-onset AF

Previous studies have implicated the genetic basis of AF and found that both common and rare variants in ion-channel genes, gap junction and transcription factor genes, or structural genes are likely associated with AF pathogenesis (16, 17).

For the ion channels, the KCNQ1 and SCN5A genes, encoding the pore-forming *α*-subunit of the cardiac potassium-channel IKs and the *α*-subunit of the cardiac sodium channel, respectively, are involved in current processes that alter the voltage dependence of channel gating, which are associated with early-onset AF. Their variants are also linked to DCM, Brugada syndrome, and ventricular fibrillation (16, 18). In addition, mutations in genes related to signalling molecules also play a role in the development of AF. It has been postulated that GATA4 and GATA5, cardiac transcription factors involved in myocardial development, directly coregulate SCN5A. GATA4, GATA5 and GATA6 are linked to decreased transcriptional activity and may play a role in reducing the levels of NKX2.5 and other target proteins, which could have further downstream effects on cardiac development and function or electrical activity (16, 17). The LMNA gene variants interact with the NUP155 gene, encoding lamin A/C and nucleoporin 155. The latter reduces nuclear envelope permeability by affecting the overall nuclear pore complex and subsequently leads to shortened action potential duration (APD), which is thought to be related to the occurrence of AF, DCM and muscular dystrophy. Myozap, a myocardial ribbon adhesion protein encoded by MYZAP, is primarily expressed in the human heart and is thought to be associated with a subtype of atrial cardiomyopathy. KLF15 is specifically expressed in myocytes and fibroblasts, playing a role in inhibiting hypertrophy and fibrosis. Li et al. found a KLF15 mutant (K229*) in a large family with AF. The affected individuals also manifested as premature ventricular contractions, and several manifested as ventricular tachycardia and HCM (17, 19).

More recently, a growing body of evidence has found that rare gene variants accounting for inherited ventricular cardiomyopathies (e.g., DCM, HCM, and ARVC) or arrhythmias (e.g., long QT syndrome) are predominantly associated with substantial AF penetrance (20). Furthermore, the identified gene variants are more often linked to inherited cardiomyopathies than arrhythmia syndrome (3, 21). Yoneda et al. (22) found that disease-associated variants in patients with early-onset AF were most frequent in genes associated with DCM (7.2%), followed by ARVC (3.3%) and HCM (2.9%). Some rare variants in genes affecting cardiac structure, such as MYH7, MYBPC3, MYL4 and TTN, have been associated with AF incidence (16). MYH7 and MYBPC3 account for more than 40% of HCM patients with pathogenic variants (23). The increased expression of MYH7 may lead to extensive myocardial disease and reduce cardiac performance, which may be related to the high occurrence of AF (16). MYL4 encodes atrial light chain-1, a protein that is expressed in foetal and adult cardiac atrial tissue. Loss-of-function variants of MYL4 can cause early atrial fibrosis, resulting in atrial cardiomyopathy and atrial arrhythmia, as well as atrial contractile failure and atrial enlargement. Titin is a giant sarcomere protein encoded by TTN, which may be associated with impaired sarcomere function caused by loss-of function variants of TTN, leading to an increased susceptibility to arrhythmia, such as early-onset AF (16, 17, 21). Vad et al. found that rare loss-of-function variants in DCM-related cytoskeletal genes (DMD, PDLIM3, and FKTN) may play a role in the development of atrial cardiomyopathy and early-onset AF (16, 17). FLNC encodes filamin-C, a cytoskeletal protein that anchors membrane proteins to the cytoskeleton in both skeletal and cardiac muscle by stabilizing polymerized actin. Variants in FLNC may present with AF, conduction disease, or ventricular arrhythmias prior to a diagnosis of cardiomyopathy (20). The SGCG gene encodes the gamma-sarcoglycan protein, and it has previously been associated with DCM. Furthermore, the SGCG gene is thought to be linked to AF in a large genome-wide association study (24). In addition, prior clinical and genetic studies indicated that pathogenic variants in other sarcomere protein genes, including TNNT2, TNNI3, TPM1, MYL2, MYL3 and ACTC1, were associated with the occurrence of ventricular and atrial arrhythmias (particularly AF) (15, 23).

Several case-control studies have demonstrated the positive association of gene variants linked to cardiomyopathies with early-onset AF (21, 24, 25). Nevertheless, whether patients with unexplained AF should be screened for cardiomyopathy-associated gene variants remains controversial (26). A recent observational prospective cohort study (22) by Yoneda et al. enrolled 1,293 patients with early-onset AF (defined as AF diagnosed at <66 years of age) and performed whole genome sequencing using the major commercial cardiomyopathy and arrhythmia-susceptibility gene panels (145 genes), identifying a disease-associated variant in 10.1% of patients. More interestingly, the authors found that the prevalence of disease-associated variants was approximately the same (10%) in the 40- to 60-year age group but up to 16.8% with AF onset before 30 years of age. These findings potentially support the use of genetic testing in early-onset AF, especially for patients before 30 years of age.

Of note, no study has evaluated the subsequent occurrence of the cardiomyopathy phenotype when a pathogenic cardiomyopathy gene variant has been identified in patients with early-onset AF. Nevertheless, after a median of 10 years of follow-up, the presence of a pathogenic or likely pathogenic variant in cardiomyopathy genes was associated with a 1.5-fold higher risk of all-cause mortality among patients with early-onset AF (27). These studies may support the use of genetic testing in early-onset AF. The consensus on how to appropriately measure the impact of genetic assessment and testing on clinical risk-benefit analyses is still developing. In addition to these limitations, the interpretation of genetic testing is extremely challenging and should be approached with caution. As larger datasets become available, it is reasonable to expect that more pathogenic variants will be discovered and provide important prognostic information for patients with early-onset AF.

## Pathophysiological mechanisms of AF remodelling in HCM

AF has been found to be secondary to an underlying atrial cardiomyopathy encompassing primary atrial disorders and secondary atrial remodelling (28). Multiple factors (i.e., environmental, clinical, and genetic) potentially lead to different pathophysiological and histological subtypes of atrial cardiomyopathy, responsible for AF vulnerability (28, 29). Genetic variants have been found to be prevalent in patients with both AF and HCM (30). However, whether HCM-associated gene variants have direct or indirect potential for AF development is not well defined. Previously, experts regularly thought that genetic variants first caused ventricular cardiomyopathy and increased left ventricle (LV) filling pressures, which subsequently led to increased atrial filling pressures, atrial stretch, and atrial dilation, ultimately causing AF development. Nevertheless, which underlying genetic HCM contributes to the atrial substrate predisposing to AF remains unclear.

More recently, the coexistence of a genetic atrial substrate in HCM patients has been increasingly considered the primary culprit of AF. This alternative hypothesis is potentially supported by several observations. Variants in atria-specific genes may be the direct drivers of AF among individuals with HCM. For instance, MYH6 encodes the *α*-subunit of myosin heavy chain predominantly expressed in atrium. Myosin, an ATPase cellular motor protein whose heavy chain subunit is a main component of the sarcomere, is the building block of the contractile system of cardiac muscle. The overexpression of MYH6 in HL-1 and isolated rat atrial cardiomyocytes results in sarcomere impairment, electrophysiological abnormalities, and a slower conduction velocity, suggesting the potential role of MYH6 gene variants in atrial structure and function. Comparable pathways may play a role in mutant MYH6-induced AF (31, 32). MYL4 is a chamber-specific expression restricted to the atria, which encodes atrial Light Chain-1, a key sarcomeric component. E17K transgenic zebrafish showed myofibrillar disarray and absent Z-disks under electron microscopy. Z-disks can form T-tubules that have a high density of LTCCs through cell membrane invaginations. The activation of LTCC triggers ryanodine receptor activation, resulting in the further release of calcium from the sarcoplasmic reticulum and subsequent sarcomere activation and contractility. Hence, the E11K-MYL4 mutation causes destabilization of the F-actin–Z-disk complex, which may impair calcium signalling and cause atrial myopathy, leading to atrial arrhythmias, especially in AF (33, 34). Among the HCM-related individual genes, it is speculated that some ventricular cardiomyopathy genes expressed in both the atria and ventricles could give rise to an atrial cardiomyopathy phenotype, subsequently manifesting as electrophysiological or structural changes affecting the atria and developing AF. For instance, TTN encodes a sarcomeric protein, titin, and is widely expressed in both the atria and ventricles. Loss-of-function variants in TTN are the most common in early-onset AF. Ahlberg et al. observed compromised assembly of the sarcomere in both the atria and ventricle, a prolonged PR interval, and a higher degree of atrial fibrosis in heterozygous adult zebrafish, suggesting that TTNtv is an important risk factor for AF (22, 25, 35–37). The next most commonly affected gene is MYH7 (22). Patients with HCM attributable to MYH7 (encoding *β*-MyHC) gene variants have a higher risk of AF than those with variants in other sarcomeric genes. Furthermore, in the early stages of HCM, genetic variation in MYH7 is related to higher levels of propeptide of type I procollagen, a marker of collagen synthesis, indicating that fibrosis can mediate a link between MYH7 and AF (3, 13). However, the underlying genetic aetiology for AF in patients with HCM is still unclear.

Examining the atrial substrate in the HCM mouse model may help explain AF development in HCM. The missense mutation Glu180Gly in the *α*-tropomyosin gene was previously detected in familial patients with HCM (38). A transgenic mouse model with the *α*-tropomyosin Glu180Gly variant was established. Compared with nontransgenic and control mice expressing wild-type *α*-tropomyosin, mutant mice at baseline presented severe biatrial remodelling and diastolic dysfunction (39–41), although whether mutant mice displayed an LV hypertrophy phenotype varied across studies (39, 41). The left atrial size of *α*-tropomyosin Glu180Gly mice was clearly larger than that of their controls (40), which is an independent risk factor for AF development. In a transgenic HCM mouse model with the cardiac troponin-I Gly203Ser variant, Lim et al. (42) first tried to assess atrial structural and electrophysiological alterations and circulating biomarkers. Compared with control mice, HCM mice with the troponin-I Gly203Ser variant showed enlarged left and right atria, increased atrial myocardial mass, significant atrial structural (myocyte hypertrophy and fibrosis) and electrophysiological (conduction) abnormalities, increased levels of blood biomarkers of extracellular matrix remodelling (MMP-2, MMP-3), and inflammation (VCAM-1). Nevertheless, Lim et al. did not document any inducible AF in the murine atrium in ex vivo electrophysiological experiments, which warrants further examination by telemetric electrocardiography in conscious animals. In addition, Pioner et al. found that HCM mouse models expressing TNNT2 variants (“hot-spot” site-R92Q and “sporadic” site-E163R) displayed atrial structural and electrophysiological remodelling. More interestingly, the pathogenesis of atrial cardiomyopathy and AF occurrence were TNNT2 variant-dependent, where E163R increased myofilament tension cost but showed no atrial arrhythmic propensity, whereas R92Q increased atrial myofilament calcium sensitivity, representing an intrinsic arrhythmogenic mechanism promoting AF. Overall, TNNT2 E163R promotes and sustains AF due to atrial cardiomyopathy induced by LV diastolic dysfunction in HCM, whereas R92Q causes AF related to the mutation itself. The sarcomere mutation-driven mechanism may help individualized treatment for AF in patients with HCM. However, although atrial myopathy may play a crucial role in providing a substrate predisposing to AF development in HCM patients, the specific molecular basis of AF occurrence caused by mutations in cardiac sarcomeric proteins is unclear.

## Anticoagulation for AF in patients with HCM

AF is common in patients with HCM and further elevates the risks of stroke and other thromboembolic events. Atrial cardiomyopathy in AF has been found to be associated with an increased stroke risk, potentially caused by atrial fibrosis-related hypocontractility, hypercoagulability, and endothelial dysfunction (43). Some genetic variants in cardiomyopathy are associated with a higher risk of early-onset AF, and tools are now becoming available to better understand and address arrhythmias in genetic cardiomyopathy, raising the prospect of therapies specific to mechanism, gene, and mutation. For instance, mavacamten is a new therapy targeted to decrease hypercontractility in HCM. It is an allosteric modulator developed to inhibit myosin ATPase activity and will soon be used to treat symptoms of outflow tract obstruction and improve the capacity to exercise in obstructive HCM. It is not clear how the specific genetic variants will affect the clinical responses to mavacamten, but studies with human induced pluripotent stem cells have shown that the ACTC1 variant responds more to mavacamten than the more common MYH7 variant (20). Furthermore, ranolazine is capable of normalizing Ca-handling in human HCM myocardium and in the ventricles of R92Q mice by blocking late sodium current, potentially reducing Ca-dependent arrhythmias in R92Q atria. Ranolazine is able to selectively inhibit peak sodium current in the atria, also destabilizing atrial reentry circuits. These observations prompt us towards further tests of ranolazine as a drug to prevent AF in selected HCM patients with a high-risk mutation (15). Similarly, SCN5A codes for the Nav1.5 channel, which is the target for sodium channel blockers, such as flecainide. KCNH2 encodes the Kv11.1 channel, which is the target for potassium channel inhibitor drugs, such as amiodarone (16). An intron-mediated TTN enhancer promotes cardiac-specific TTN expression in similarly derived cardiomyocytes, thus promoting normal TTN expression in mice. In addition, adenoviral-mediated modulation of RNA splicing with an HCM-related MYBPC3 mutation in induced pluripotent stem cells is able to correct the hypertrophic phenotype. Danon disease, an HCM phenotype, has been reported for 3 patients with the first-in-human gene transfection of the LAMP2 gene. The rationale to expand genetic testing extends from current management of patients and families to accelerate an exciting future. Tailored preventive treatments for AF can be identified in selected genotyped HCM subgroups to develop novel first-in-class agents that target specific molecular mechanisms in cardiomyopathy subtypes, correcting their underlying molecular defects and reducing the incidence of AF in HCM.

The HA/ACC Guideline recommends that, in patients with HCM and subclinical AF detected by internal or external cardiac device or monitor of >24 h’ duration for a given episode, anticoagulation is recommended with direct-acting oral anticoagulants (NOACs) as a first-line option and vitamin K antagonists (VKAs) as a second-line option, independent of CHA2DS2-VASc score (44). Hence, early detection of AF is extremely crucial to recognize and treat AF in a timely manner. In conclusion, we recommend close and thorough investigations using ECG, Holter ECG and implantable loop recorders on a regular basis in HCM patients at high risk for AF. As such, every HCM patient with documented AF should be immediately given lifelong oral anticoagulant treatment because AF is necessary for patients with HCM regardless of the CHA2DS2-VASc score.

Beyond VKAs, several observational studies have assessed the effect of nonvitamin K NOACs compared with VKAs in HCM patients with AF (45–49). Among the published studies, a study in the US commercial insurance database showed that NOACs use in HCM patients with AF was associated with a lower risk for ischaemic stroke and bleeding after a mean follow-up of 0.56 years compared with warfarin use (48). Data from patients with AF and HCM from the Korean National Health Insurance Service database showed that, compared with those with VKAs, patients with NOACs had significant reductions in the risks of all-cause mortality and composite fatal cardiovascular events during a median follow-up of 16 months (46). Other data from the Korean Health Insurance Review and Assessment Service database showed that the use of NOACs vs. VKAs significantly decreased the risks of ischaemic stroke and the composite outcome during 1.6 years of follow-up (45). A subsequent systematic review by Rujirachun et al. including published articles observed that the use of NOACs vs. VKAs showed a significantly lower risk of all-cause death in HCM patients with AF, but the risks of ischaemic stroke, major bleeding and intracranial bleeding were not significantly different (50). In addition, Zhou et al. compared the effect of NOACs with VKAs in patients with HCM and AF, and they found that the use of NOACs was associated with reduced risks of ischaemic stroke, all-cause death, and intracranial haemorrhage (51). In sum, these data support the notion that, compared with VKA use, the use of NOACs showed similar or lower risks of thromboembolic and bleeding events in HCM patients with AF (50–52). In addition, NOACs at least have similar effects as VKAs in patients with HCM undergoing catheter ablation for AF (53). Further head-to-head randomized clinical trials in this population could confirm the use of NOACs. It is also not known whether there are differences in anticoagulation effects regarding positive vs. negative genotypes in patients with HCM and AF. Further studies could explore whether the HCM genotype affects treatment with anticoagulants in this specific population.

## Conclusions and further implications

Recent evidence has suggested that AF could be the first presentation of genetic HCM patients in the absence of a cardiomyopathy phenotype. However, data are currently limited as to whether genetic testing should be performed in this population with early-onset AF. After sarcomere gene variants are identified in an individual with AF, the question regarding the association of sarcomere gene variants with HCM occurrence in the future also remains unanswered. Whether a cardiomyopathy variant directly or indirectly leads to the incidence of AF remains controversial and needs further examination. Moreover, how the identification of these sarcomere gene variants should influence clinical care (e.g., anticoagulation therapy) for a patient with early-onset AF is still undefined.
